# Chondrogenic Potential of Pellet Culture Compared to High-Density Culture on a Bacterial Cellulose Hydrogel

**DOI:** 10.3390/ijms21082785

**Published:** 2020-04-16

**Authors:** Nele Pascale Grigull, Julia Isabelle Redeker, Bärbel Schmitt, Maximilian Michael Saller, Veronika Schönitzer, Susanne Mayer-Wagner

**Affiliations:** 1 Department of Orthopaedics, Physical Medicine and Rehabilitation, University Hospital, LMU Munich, Marchioninistr. 15, 81377 Munich, Germany; Nelegrigull@posteo.de (N.P.G.); Julia.Redeker@icloud.com (J.I.R.); Baerbel.Schmitt@med.uni-muenchen.de (B.S.); 2 Experimental Surgery and Regenerative Medicine (ExperiMed), Department of General, Trauma and Reconstructive Surgery, Ludwig-Maximilians-University (LMU), Fraunhoferstraße 20, 82152 Martinsried, Germany; Maximilian.Saller@med.uni-muenchen.de (M.M.S.); Veronika.Schoenitzer@med.uni-muenchen.de (V.S.)

**Keywords:** cartilage repair, bacterial cellulose hydrogel, human cartilage, chondrogenic potential

## Abstract

Cell-based approaches of cartilage lesions use different culture systems to obtain optimal cell quality. Pellet cultures with high cellular density (HD) are the gold standard to keep chondrocytes in a differentiated stage. Bacterial cellulose (BC) hydrogel is discussed to prevent cellular aging and dedifferentiation. The hypothesis of this study was that HD culture on BC hydrogel (HD hydrogel) might reach the chondrogenic potential of pellet culture (pellet). Human articular osteoarthritic (OA) and non-osteoarthritic (non-OA) chondrocytes were cultured for seven days within pellets and compared to HD hydrogel and HD polystyrene. Gene expression analysis and histological assessment were performed. We observed no significant change of *COL2A1* expression by the culture system (pellet, HD hydrogel and HD polystyrene) but a significant change of *COL2A1/COL1A1*-ratio, with the highest ratio in pellets. Chondrocytes on HD hydrogel showed an elevated expression of *MMP13* and on polystyrene an increased expression of *COL1A1* and *MMP13*. The patterns of gene expression changes observed in OA and non-OA chondrocytes in reaction to the different culture systems were similar in those two cell groups. Pellet cultures moreover formed a histomorphologically superior neocartilage. Concluding, human chondrocytes kept the potential to express *COL2A1* in all HD culture systems. However, pellets excelled in a higher *COL2A1/COL1A1*-ratio, a higher extracellular matrix deposit and in not developing degeneration and dedifferentiation markers. This underlines the superiority of pellet culture in maintaining the chondrogenic potential of human chondrocytes in vitro.

## 1. Introduction

Most approaches of regenerative medicine in cartilage lesions depend on the implantation of highly differentiated chondrogenic cells. To gain enough cellular material before re-implantation in vitro, expansion of isolated cells is essential. Dedifferentiation during in vitro expansion of human chondrocytes is an obstacle, which is not overcome yet. Thus, the improvement of chondrocytes culture conditions is necessary in order to generate better tissue-engineered cartilage products.

Beneficial effects on chondrogenesis have been shown for high density (HD) and three dimensional (3D) culture systems (providing intensive cell–cell contacts) [1,2,3], alternative culture surfaces such as hydrogels (addressing cell–surface contacts) [4], and various physical stimuli (e.g., electromagnetic fields of a certain spectrum [5], or hydrostatic pressure and perfusion [6]) as well as chemical stimuli [7].

When cultured within 3D systems, chondrocytes have been shown to be even able to redifferentiate [8]. HD culture and extensive cell–cell contacts are postulated to induce the high chondrogenic potential of 3D culture [9]. 3D pellet culture systems, which are known as gold standard in the field of 3D cultures [10], have even been approved for clinical application to restore cartilage defects [chondrosphere^®^, co.don AG, Teltow, Germany]. However, pellet culture conditions with relatively small pellet sizes and the difficulty of anchorage make it obvious to further search for other alternatives.

As cartilage contains high amounts of water [11], hydrogels are promising in mimicking the natural aqueous chondrocyte environment during in vitro expansion [12]. An appropriate substratum, which is known to conserve the chondrogenic potential of cells [13], might also lead to an improvement of cartilage replacements. Although 3D pellet culture systems are understood as superior for promoting a chondrogenic phenotype [2], HD culture also offers the opportunity to regain cells for the future transfer onto extracellular matrices and implants. Providing a different cell–surface interaction than is commonly used in polystyrene culture plates, hydrogels are also considered for a HD culture system, where chondrocytes benefit from the appropriate surface [4].

Hydrogels made of bacterial cellulose (BC) are especially promising materials for tissue engineering, as they are biocompatible, provide good mechanical properties, and have a high-water binding capacity [12]. An obstacle of the utilization of BC in vivo has been the lack of biodegradability. Meanwhile, approaches have been made to overcome this limitation, e.g., to use a lysozyme degradable modified BC [14] or electron beam irradiation before implantation, which allows biodegradation of BC [15]. BC hydrogels used in this study has been reported to reduce proliferation in human umbilical vein endothelial cells, as cells are entering a quiescent state [16]. Similar successes have also been reported for other BC hydrogels in guiding regeneration of other cell types and tissues [12,17]. Even for chondrocytes, promising results with BC hydrogels supporting their redifferentiation have been shown [18,19,20,21]. Redifferentiation is always defined by an increase of cartilage specific *COL2A1* expression and a decrease of fibroblast like *COL1A1* expression, commonly put in relation to one another by *COL2A1/COL1A1*-ratio. However, evidence is missing so far concerning the comparison of BC hydrogels to the gold standard pellet culture.

In order to obtain optimal results, conditions similar to pellet culture might induce comparable results in other culture systems. Using HD culture has been shown to improve chondrogenesis up to a certain degree [22,23,24]. Although the HD culture is a two-dimensional culture form, the higher cellular density makes it possible to regain differentiation of chondrogenic cells. In combination with the aqueous surrounding of a BC hydrogel, this might lead to culture conditions approaching 3D pellets. To validate the chosen cell density for the HD experiment, demonstrate the effect of cell density, and verify the hypothesis that low cell density will lead to reduced chondrogenic potential, low-density conditions were used in the run-up to this study.

In order to gain information about osteoarthritic (OA) and non-osteoarthritic (non-OA) human articular chondrocytes, which might both be used in tissue engineering procedures, the two cell types were analyzed separately.

We hypothesized that the combination of HD with an aqueous surrounding of a BC hydrogel might induce beneficial effects on chondrogenesis, which ultimately might imitate an established 3D culture system like pellet culture. The seeding density, besides 3D culture and hypoxic culture conditions, has shown the ability to improve the in vitro culture of chondrocytes [25] and cause positive effects in autologous chondrocyte implantation [26].

The aim of this study was to compare the effect of HD in vitro culture on BC hydrogel or polystyrene with pellet culture of human OA and non-OA articular chondrocytes in respect to their gene expression levels of chondrogenic markers, as well as to their proteoglycan deposit in their extracellular matrix (ECM). The clinical use of BC hydrogels would, also because of its 2D culturing form, be a clear alternative to the pellet culture system with its excellent chondrogenic potential but relatively difficult handling.

## 2. Results

Human OA and non-OA chondrocytes of passage 2, 3, and 4 were cultured in HD culture on polystyrene, BC hydrogel or in pellet culture, respectively. After 7 days in culture, cells were harvested and mRNA-analysis and histological assessment were performed.

### 2.1. Histology and Visual Grading

**Figure 1:**
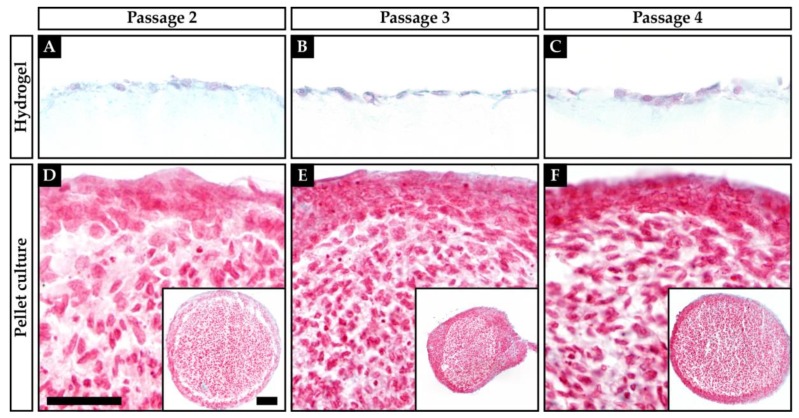
Safranin O/Fast Green staining of non-osteoarthritic (non-OA) human articular chondrocytes growing on a hydrogel (**A**–**C**) or in pellet culture (**D**–**F**). (**A**+**D**) Passage 2; (**B**+**E**) Passage 3; (**C**+**F**) Passage 4. Scale bars: 50 µm (**A**–**F**), 100 µm (**D**–**F**, inserts).

**Figure 2:**
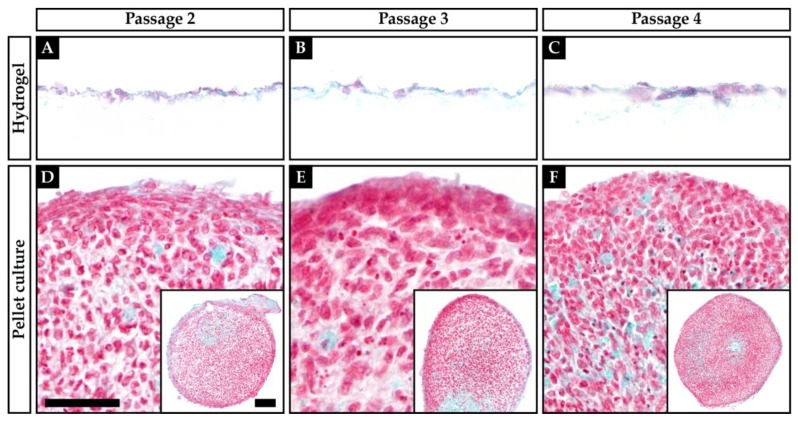
Safranin O/Fast Green staining of osteoarthritic (OA) human articular chondrocytes growing on a hydrogel (**A**–**C**) or in pellet culture (**D**–**F**). (**A**+**D**) Passage 2; (**B**+**E**) Passage 3; (**C**+**F**) Passage 4. Scale bars: 50 µm (**A**–**F**), 100 µm (**D**–**F**, inserts).

Both pellet culture and chondrocytes cultured on hydrogel showed Safranin O-stained ECM (Figure 1 and Figure 2). In pellet culture, ECM was spread between the cells, whereas on hydrogel the ECM deposit was rather pericellular. The staining showed a higher intensity in pellet culture, and the cell morphology was rounder and more cartilage-like when compared to hydrogel culture. The Bern score [27] rates Safranin O staining, cell morphology, and cell–cell distance. The best possible score is nine points, indicating a good cartilage-like quality of the tissue, whereas a low score indicates a poor quality of the tissue. Throughout all passages, the pellet-cultured chondrocytes of both OA and non-OA formed a better neocartilage, when compared to chondrocytes cultured on the hydrogel matrix, resulting in higher scores for the pellet cultured chondrocytes (Table 1).

Concluding, the pellet-cultured chondrocytes of both OA and non-OA throughout all passages appear to form a better neocartilage than chondrocytes cultured on the hydrogel matrix.

### 2.2. Gene Expression

Expression levels of markers of chondrogenic differentiation (*COL2A1*, *SOX9*, *ACAN*), chondrogenic dedifferentiation (*COL1A1*) and matrix catabolism (*MMP13*) were analyzed by qPCR.

**Figure 3:**
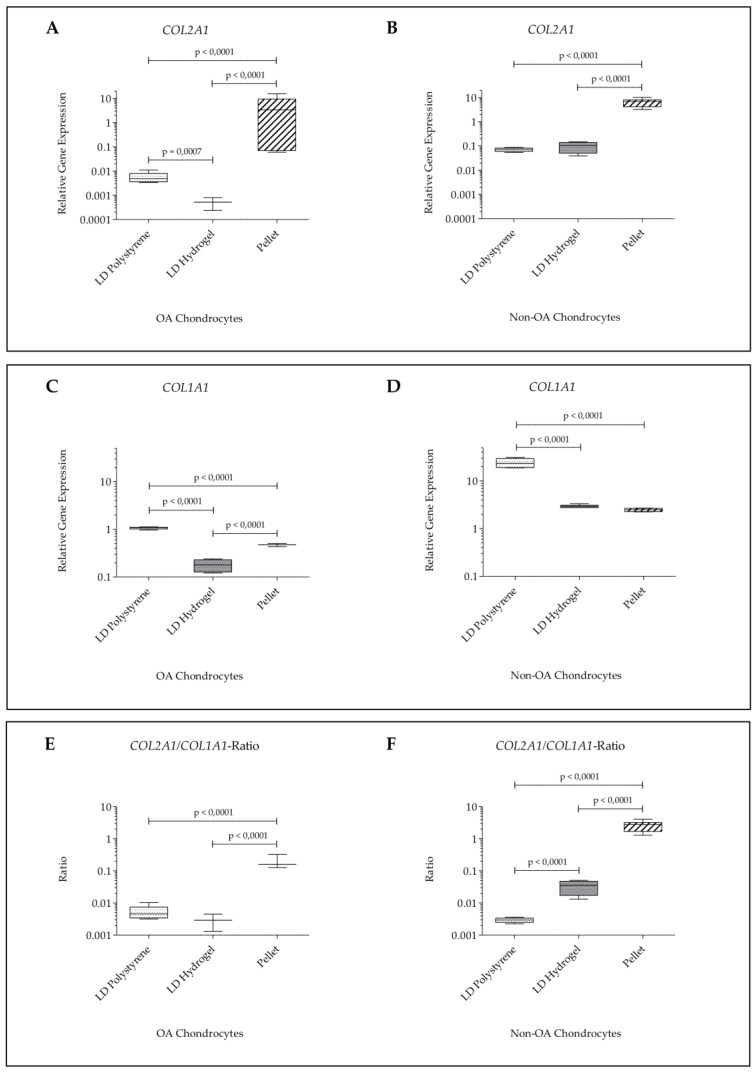
Validation experiments with low seeding density: relative gene expression normalized to *GAPDH* and day 0 (2^−∆∆*C*t^) of human articular chondrocytes after 7 days of culture in passage 2 on hydrogel, on polystyrene or in pellet culture. (**A**) *COL2A1* expression in OA chondrocytes; (**B**) *COL2A1* expression in non-OA chondrocytes; (**C**) *COL1A1* expression in OA chondrocytes; (**D**) *COL1A1* expression in non-OA chondrocytes; (**E**) *COL2A1/COL1A1*-ratio in OA chondrocytes; (**F**) *COL2A1/COL1A1*-ratio in non-OA chondrocytes.

In order to validate the chosen cell density for the HD experiments, a low cell density of 5 × 10^4^ cells/cm^2^ on polystyrene or on hydrogel was evaluated, compared to pellet cultures and examined for expression levels of *COL2A1* and *COL1A1*. In these experiments, *COL2A1* expression and the *COL2A1/COL1A1*-ratio were significantly reduced in low-density (LD) cultures on polystyrene and on hydrogel of OA and non-OA chondrocytes compared to pellet cultures (Figure 3).

**Figure 4:**
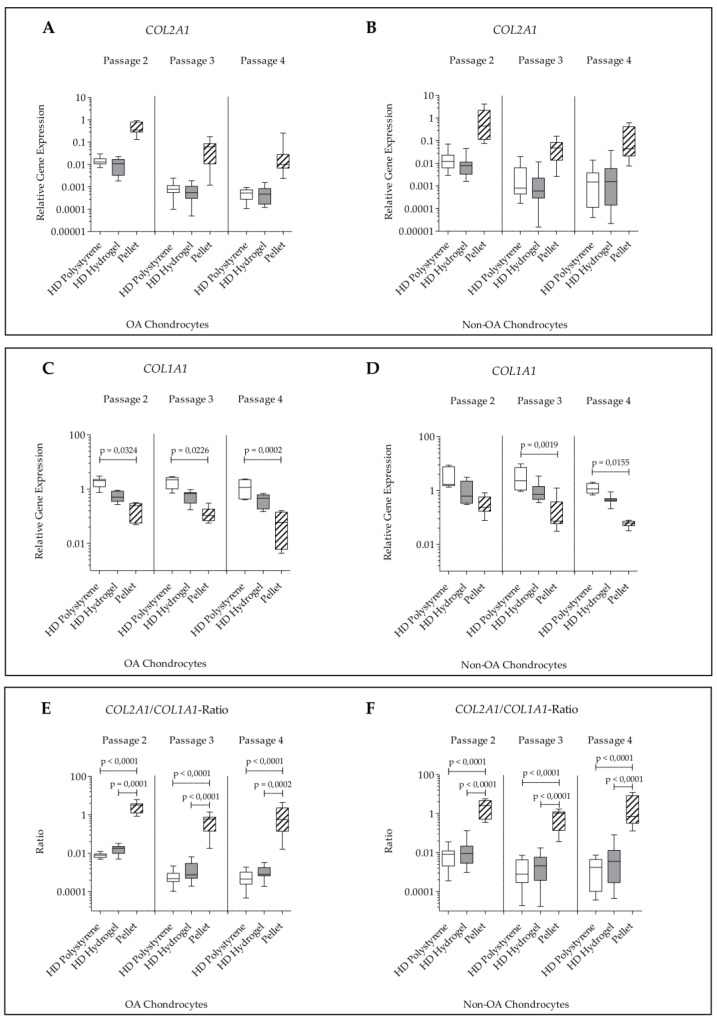
Relative gene expression normalized to *GAPDH* and day 0 (2^−∆∆*C*t^) of human articular chondrocytes after 7 days of culture in passage 2, 3, and 4 on hydrogel, on polystyrene or in pellet culture. (**A**) *COL2A1* expression in OA chondrocytes; (**B**) *COL2A1* expression in non-OA chondrocytes; (**C**) *COL1A1* expression in OA chondrocytes; (**D**) *COL1A1* expression in non-OA chondrocytes; (**E**) *COL2A1/COL1A1*-ratio in OA chondrocytes; (**F**) *COL2A1/COL1A1*-ratio in non-OA chondrocytes.

The experiments with high cell densities showed that the type of culture (pellet, HD hydrogel and HD polystyrene) had no significant effect on *COL2A1* expression of non-OA and OA chondrocytes throughout all passages (Figure 4A,B).

*COL1A1* expression within pellet cultures from OA chondrocytes from all passages was significantly lower than *COL1A1* expression of HD culture on polystyrene. In non-OA pellet cultured chondrocytes, a significant lower *COL1A1* expression compared to HD culture on polystyrene was observed for passage 3 and 4. The *COL1A1* expression levels were not significantly changed in HD culture on polystyrene and HD culture on hydrogel independent of passage and cell type (OA chondrocytes, non-OA chondrocytes) (Figure 4C,D).

The *COL2A1/COL1A1*-ratio was significantly increased in pellet cultures of OA and non-OA chondrocytes compared to HD culture on polystyrene and on hydrogel throughout all passages. No significant difference of *COL2A1/COL1A1*-ratio was seen between HD culture on polystyrene and on hydrogel independent of passage and cell type (Figure 4E,F).

**Figure 5:**
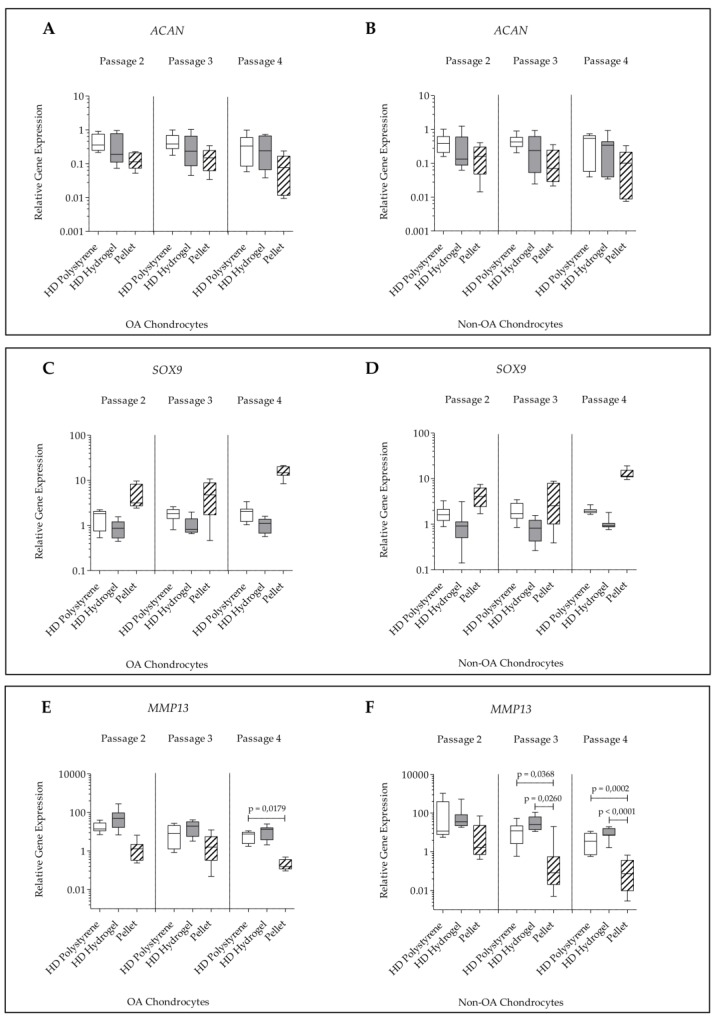
Relative gene expression normalized to *GAPDH* and day 0 (2^−∆∆*C*t^) of human articular chondrocytes after 7 days of culture in passage 2, 3, and 4 on hydrogel, on polystyrene or in pellet culture. (**A**) *ACAN* expression in OA chondrocytes; (**B**) *ACAN* expression in non-OA chondrocytes; (**C**) *SOX9* expression in OA chondrocytes; (**D**) *SOX9* expression in non-OA chondrocytes; (**E**) *MMP13* expression in OA chondrocytes; (**F**) *MMP13* expression in non-OA chondrocytes (non-OA pellet passage 2 compared to non-OA pellet passage 4: *p* = 0.0002).

For *ACAN* and *SOX9* expression, no significant changes were detected within all culture conditions (pellet, HD hydrogel, and HD polystyrene) independent of passage and cell type (Figure 5A–D).

For *MMP13* a significantly higher expression was found in HD culture on polystyrene at passage 4 in comparison to pellet cultures of OA chondrocytes of the same passage. HD culture on polystyrene and HD culture on hydrogel did not differ significantly in their *MMP13* expression levels, independent of passage. In non-OA chondrocytes, the *MMP13* expression was found to be significantly elevated in HD culture on polystyrene and HD culture on hydrogel compared to pellet cultures in passage 3 and 4 (Figure 5E,F).

Altogether, comparing the changes in gene expression of OA and non-OA chondrocytes in reaction to the different culture forms (pellet, HD hydrogel and HD polystyrene), similar patterns of gene expression changes were observed. Chondrocytes of the same culture form showed tendencies of decreased *COL2A1* expression and *COL2A1/COL1A1*-ratio within passages.

## 3. Discussion

As it is known that matrix systems already used in matrix-associated chondrocyte transplantation (MACT) do not reach the differentiation level of native cartilage [28], the importance of new approaches and their comparison to well established systems is obvious.

Using BC hydrogels as scaffolds has been discussed, due to the excellent biocompatibility and tissue integration capability [18]. Various BC hydrogels of aberrant morphometry have been described to support cartilage formation [18,19]. For BC hydrogels, the chemically unmodified form has been described to be the most suitable for chondrocyte growth [20].

Cells interact with the scaffolds they are seeded on and scaffolds imitating ECM contacts influence cell differentiation [29]. In BC hydrogels, collagen fibrils usually form a very dense mesh with few micrometers of pore size. These are too small to allow cell ingrowth, but support the formation of collagen fibers and ECM [12]. BC has been examined as hydrogel matrix for the redifferentiation of bovine and human chondrocytes and the chondrogenic differentiation of human mesenchymal stem cells [18,19,20,21,30]. BC hydrogel has been proven suitable to cultivate nasoseptal chondrocytes for a long time [18].

However, a novel culture method should always be compared to gold standards in the field. Here, data for BC hydrogels are missing, since there is, to our knowledge, no comparative study showing BC hydrogel cultures compared to the pellet culture system.

Pellet culture is a scaffold free 3D culture form, which is commonly used to stabilize the chondrogenic potential of in vitro cultured chondrocytes [2,7,31]. This is mainly due to the high cellular density within pellet culture inducing a high amount of cell–cell contacts.

As expected, our experiments with reduced cell density showed that a low cell density of 5 × 10^4^ cells/cm^2^ on polystyrene or on hydrogel significantly reduced *COL2A1* expression and the *COL2A1/COL1A1*-ratio of OA and non-OA chondrocytes compared to pellet cultures. Therefore, all further experiments were performed within HD culture. It was hypothesized that human chondrocytes would profit from being cultured in the combination of HD culture and BC hydrogel.

In the combination setting, *COL2A1* expression was found not to be significantly altered within the different culture systems (pellet, HD hydrogel, and HD polystyrene). However, the *COL2A1/COL1A1*-ratio was found significantly higher in pellet-cultured chondrocytes compared to BC hydrogel and polystyrene cultures. In comparison to pellet culture, there was a partly significant elevation of *MMP13* in chondrocytes cultured at HD on BC hydrogel. Chondrocytes cultured at HD on polystyrene even showed significant elevations of *COL1A1,* as well as *MMP13*, when compared to pellet culture.

As hydrogels can be optimized in many ways, including the electrical charge density [4], porosity [21], layering [18], structure [19], and other factors, this might explain the suboptimal results reached with the unmodified BC hydrogel used in this study. Nevertheless, we chose the non-optimized hydrogel properties in order to concentrate on the HD effect, which is one of the main factors leading to chondrogenic differentiation [3]. It appears that the most obvious benefit for the chondrogenic potential resulted from the HD culture. All three culture groups (pellet, HD hydrogel, and HD polystyrene) expressed mRNA of the main chondrogenic protein collagen II without statistically significant differences to be detected. As many studies compare the chondrogenic potential of matrices at lower cell densities, this might produce misleading results paying high attention to surface properties. Further studies on BC hydrogels might be needed to also examine the influence of cell densities, as this is known to be the essential factor increasing the chondrogenic potential.

Recent studies have shown the suitability of OA chondrocytes for tissue engineering approaches [32,33], as the chondrogenic capacity is not turned off by OA [31]. In this study, similar changes in gene expression were observed for OA and non-OA chondrocytes in reaction to the different culture forms. It might have been expected that the HD effect would preponderate in one of the groups, but within this study both OA and non-OA chondrocytes profited by HD culture. This underlines the possible use of OA chondrocytes for regenerative approaches, as OA chondrocytes seem to be able to react to redifferentiation stimuli.

Redifferentiation potential of chondrocytes at higher passages is discussed [1,23]. It already has been postulated that cell-seeding density has a higher impact on chondrogenic potential than the stage of dedifferentiation before HD culture [3]. Within this study, a tendency of a decrease of *COL2A1* expression and the *COL2A1/COL1A1*-ratio in all culture forms was observed at higher passages. However, no significant changes in gene expression due to passage were found, indicating that all groups could redifferentiate to a certain degree in HD culture up to passage 4. This further enforces the theory that seeding density can outrun dedifferentiation up to a certain degree and will play a similar major role for BC hydrogels.

The proteoglycan deposit in the ECM of the chondrocytes in hydrogel and pellet culture was examined by histology. Although, there was a positive Safranin O staining of both cultures, pellet cultures achieved a higher Bern score than chondrocytes on hydrogel, indicating a better quality of the neocartilage formation. In pellet culture, ECM spread wider between the cells than on hydrogel, where the ECM deposit was rather pericellular. The Bern score is a useful tool to assess the histologic staining, although it might be affected by the different culture situations. Limitation of this study was the lack of a three-dimensional cell presentation within the HD culture on the BC hydrogel, where only a two-dimensional rating was performed. Immunohistological proof, which might have delivered more information about protein presentations, failed in the BC experiments (data not shown) as the background staining within BC hydrogels is too strong.

Regarding the further limitations of this study, a very short cultivation period of seven days was chosen. However, this short period was used to deliver results suitable for the short timeframes of adjuvant therapies, which are necessary in order to prevent long immobilization, where chondrogenic potential is lost.

Despite the limitations of the study, it can be summarized that both OA and non-OA human articular chondrocytes retained their chondrogenic potential at high seeding density within all culture conditions (pellet, HD hydrogel, and HD polystyrene). Nevertheless, the pellet culture system excelled in suppressing degeneration and dedifferentiation markers of chondrocytes and showed a higher ECM deposit.

## 4. Materials and Methods

### 4.1. Bacterial Cellulose (BC) Hydrogel Matrix

**Figure 6:**
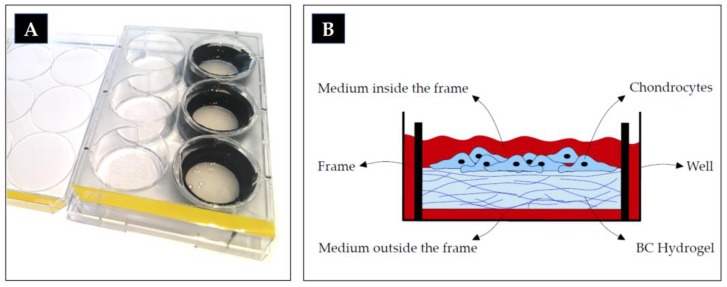
6-well culture plate; in the right column filled with the bacterial cellulose (BC) hydrogel mounted on black frames (**A**); schematic diagram of a cross section of a well in cell culture (**B**).

The morphologically characterized BC hydrogel Xellulin^®^ (Xellutec, Neuried, Germany) [34] was synthesized by Gluconacetobacter xylinus [16] containing approximately 99% of water. It was mounted on a round frame and placed in a 6-well cell culture plate with medium underneath and on top of the hydrogel, where the cells were seeded (Figure 6). The hydrogel was shipped freshly in phosphate buffered saline (PBS), which was replaced 24 h prior to cell seeding with chondrocyte medium containing 20% of fetal calf serum (FCS).

### 4.2. Cell Culture

OA chondrocytes were obtained from human OA articular cartilage from patients undergoing a total knee replacement (n = 4; all females; mean age 67.5 years; range 62–73 years). In order to compare OA chondrocytes to the best possible cartilage quality, the non-OA chondrocytes were not age matched but instead obtained from knees of young deceased persons without apparent joint disease, up to 12 h after death (n = 4; 3 males, 1 female; mean age 25.5 years; range 20–29 years; Institute for Legal Medicine, Ludwig-Maximilians-University). The study was approved by the responsible medical center ethics committee (No 69-16, Ludwig-Maximilians-University). Chondrocytes were isolated by enzymatic treatment (0.5% pronase for 1 h at 37 °C [Roche, Basel, Switzerland], 0.1% collagenase for 4 h at 37 °C [Sigma-Aldrich, St. Louis, USA]) and cultured in monolayer at 37 °C in a humidified atmosphere in chondrocyte medium (DMEM/Ham’s F-12 [Biochrom, Berlin, Germany] with ascorbic acid 25 µg/mL [Sigma-Aldrich, St. Louis, MO, USA], Mem amino acids 1% [Thermo Fisher Scientific, Waltham, MA, USA], 50 IU/mL penicillin/streptomycin [Biochrom, Berlin, Germany], amphotericin B 0.25 mg/mL [Sigma-Aldrich, St. Louis, MO, USA] and FCS 10% [Biochrom Berlin, Germany] as supplements) for one, two, or three passages respectively, according to Redeker et al. [5].

In order to validate the chosen cell density for the HD experiments, a low cell density was evaluated in a shortened experimental setup: passage 2 chondrocytes with a seeding density of 5 × 10^4^ cells/cm^2^ on polystyrene and hydrogel 6-well-plates were compared to pellet cultures (4 × 10^5^ cells/pellet). This was performed to demonstrate the effect of cell density and verify the hypothesis that low cell density will lead to reduced chondrogenic potential.

For HD cultures, chondrocytes were seeded at a density of 10^5^ cells/cm^2^ on polystyrene or on hydrogel 6-well-plates. For pellet cultures, 4 × 10^5^ cells were centrifuged for 5 min at 150 g in 15 mL polystyrene tubes. Medium was changed every other day.

OA and non-OA chondrocytes were cultured in HD culture either on polystyrene or on a BC hydrogel or in pellet culture. All groups were cultured for 7 days under the same conditions at passages 2, 3, and 4, respectively. At the end of the cultivation period, the cells were harvested for mRNA-analysis and embedded for histological analysis.

### 4.3. Histology

The medium was removed, and pellets and the hydrogel plates were washed with PBS (Biochrom, Berlin, Germany). Then, a slice of hydrogel was cut with a scalpel from the widest diameter of the well from each group.

Pellets and hydrogel were incubated in 5% sucrose (Sigma-Aldrich, St. Louis, MO, USA) in PBS (21 °C, 15 min), dry embedded in Tissue-Tek O.C.T. Compound (Sakura Finetek, Tokyo, Japan), and stored at −20 °C. Serial sections (10 µm) were prepared and the material was mounted, dried, and fixated with acetone.

Sections were stained with 0.75% Safranin O (Morphisto, Frankfurt, Germany) and 0.02% Fast Green (Morphisto, Frankfurt, Germany).

### 4.4. Visual Histologic Grading System

For histological analysis, the Bern score was used as described in [27]. Shortly, three independent and blinded observers evaluated the sections, scoring Safranin O staining, cell morphology, and cell–cell distance with 0 to 3 points for each criterion. A high score indicates a good cartilage like quality of the tissue, whereas a low score indicates a poor quality of the tissue. Table 1 displays the mean score of the three observers for each criterion.

### 4.5. RNA Extraction

For RNA isolation, cells were lysed using Trizol reagent (Thermo Fisher Scientific, Waltham, MA, USA). Total RNA was purified by Trizol-chloroform extraction. RNA precipitation was performed using isopropanol. RNA pellets were washed with ethanol and the dried pellets were resuspended in RNAse free water. RNA amount and purity were measured with the NanoDrop Lite Spectrophotometer (Thermo Fisher Scientific).

### 4.6. Quantitative Real-Time Polymerase Chain Reaction (q-PCR)

For qPCR analysis, cDNA synthesis was performed using the Quanti Tect Reverse Transcription Kit (Qiagen, Hilden, Germany) as recommended by the producer. All qPCR experiments were done in triplicates using the LightCycler 96 (Roche, Basel, Switzerland) and Faststart Essential DNA Green Master (Roche, Basel, Switzerland) according to manufacturer instructions and adapted to the primers (Metabion, Planegg, Germany) used (Table 2).

### 4.7. Statistical Analysis

Mean relative quantification values were calculated by the 2^−ΔΔCT^ method [40] using *GAPDH* as an endogenous control. In this study, day 0 of chondrocyte monolayer culture was used as calibrator in the 2^−ΔΔCT^ method for all samples. This commonly used evaluation [40] allows us to compare the expression levels of the markers in the different culture conditions (pellet, HD hydrogel, and HD polystyrene). Statistical analyses relied on mixed linear models, fitting a random intercept for each patient, using the decadic logarithm of the relative gene expression data as dependent variables. These analyses were performed using the MIXED procedure of the Statistical Analysis System SAS, version 9.4 for Windows (SAS Institute, Cary, NC, USA). In the graphs, box and whiskers plots were used to display the PCR data (boxes show the 25th to 75th percentiles; middle lines show the medians; whiskers show minimum to maximum). The statistical significance is shown in the graphs with the Bonferroni adjusted *p*-value. Each box in the graphs displays the qPCR data of four independent experiments (three experiments in passage 4), consisting of two independent RNA samples of each group and experiment, all analyzed in triplicates in qPCR.

## 5. Conclusions

In this study, a combination of HD culture and BC hydrogel in comparison to pellet culture of human articular chondrocytes was evaluated. At high cellular densities, *COL2A1* expression was found not to be significantly altered by the culture system (pellet, HD hydrogel, and HD polystyrene), whereas the *COL2A1/COL1A1*-ratio was measured highest in pellet cultured chondrocytes. Chondrocytes on hydrogel showed a mixed phenotype with partly elevated expression of *MMP13*, whereas chondrocytes on polystyrene showed elevations of *COL1A1* and *MMP13*. The patterns of gene expression changes observed in OA and non-OA chondrocytes in reaction to the different culture forms (pellet, HD hydrogel and HD polystyrene) were similar in those two cell groups. It can be summarized that human articular chondrocytes display their chondrogenic potential best in pellet culture, underlining the superiority of this 3D culture system in maintaining the chondrogenic differentiation when culturing human chondrocytes in vitro.

## Figures and Tables

**Table 1:** Bern Score of each criterion (A = intensity of Safranin O staining, B = cell morphology, C = cell–cell distance).

Score	Culture	Passage	A	B	C	Sum
OA	Hydrogel	2	1	2	2	5
		3	1	2	2	5
		4	1	2	2	5
	Pellet	2	2	3	3	8
		3	2	2	3	7
		4	2	3	3	8
Non-OA	Hydrogel	2	1	2	1	4
		3	1	2	2	5
		4	1	2	2	5
	Pellet	2	2	3	3	8
		3	2	3	3	8
		4	2	3	3	8

**Table 2:** Used primers with sequences and references.

Gene	Primer Sequence		Reference
*GAPDH*	forward	TGCACCACCAACTGCTTAGC	[35]
	reverse	GGCATGGACTGTGGTCATGAG	
*COL2A1*	forward	GTTATCGAGTACCGGTCACAGAAG	[36]
	reverse	AGTACTTGGGTCCTTTGGGTTTG	
*ACAN*	forward	CAGCACCAGCATCCCAGA	[36]
	reverse	CAGCAGTTGATTCTGATTCACG	
*COL1A1*	forward	TGACCTCAAGATGTGCCACT	[37]
	reverse	ACCAGACATGCCTCTTGTCC	
SOX9	forward	AGACCTTTGGGCTGCCTTAT	[38]
	reverse	TAGCCTCCCTCACTCCAAGA	
*MMP13*	forward	GACTTCACGATGGCATTGCTG	[39]
	reverse	GCATCAACCTGCTGAGGATGC	

## Abbreviations

3D	three dimensional
*ACAN*	Cartilage-specific proteoglycan core protein
BC	bacterial cellulose
*COL1A1*	Collagen type I alpha 1 chain
*COL2A1*	Collagen type II alpha 1 chain
DMEM	Dulbecco’s modified eagle’s medium
ECM	extracellular matrix
FCS	fetal calf serum
*GAPDH*	Glycerinaldehyd-3-phosphat-dehydrogenase
HD	high density
LD	low density
MACT	matrix-associated chondrocyte transplantation
*MMP13*	matrix metalloproteinase 13
non-OA	non-osteoarthritic
OA	osteoarthritic
p	*p*-value, probability
PBS	Phosphate buffered saline
qPCR	Quantitative real-time polymerase chain reaction
*SOX9*	SRY (sex determining region Y)-box 9
